# Endoscopic carpal tunnel release surgery: a case study in Vietnam

**DOI:** 10.1186/s13018-019-1192-z

**Published:** 2019-05-24

**Authors:** Dung Tran Trung, Thanh Ma Ngoc, Du Hoang Gia, Son Dinh Ngoc, Son Le Manh, Toan Duong Dinh, Thanh Dao Xuan, Minh Do Van, Long Nguyen Hoang

**Affiliations:** 1 Hanoi Medical University, No 1, Ton That Tung Street, Hanoi, 10000 Vietnam; 2 Bach Mai University Hospital, 78 Đường Giải Phóng, Phương Đình, Đống Đa, Hanoi, 10000 Vietnam; 3 VietDuc University Hospital, 40 Tràng Thi, Hàng Bông, Hoàn Kiếm, Hanoi, 10000 Vietnam

**Keywords:** Carpal tunnel syndrome, Single portal endoscopic

## Abstract

**Background:**

This study aims at investigating the outcome and electrophysiologic recovery of 150 carpal tunnel syndrome hands after single-portal endoscopic surgery.

**Methods:**

Patients with the cross-sectional area of the median nerve being 13–15 mm^2^ on ultrasound or abnormal sensory nerve conduction velocity on EMG are assigned to endoscopic surgery that cuts the decompressing transverse ligament to avoid the emergence of severe symptoms, such as muscular atrophy and loss of hand function.

**Results:**

Single-portal endoscopic release is a safe and efficacious option for carpal tunnel release. The findings demonstrate encouraging results.

**Conclusion:**

The endoscopic carpal tunnel release with the placement of a MicroAire system is a safe and effective method for treating carpal tunnel syndrome.

## Introduction

The carpal tunnel syndrome (CTS) is a common problem that affects the hand and wrist. The syndrome results from compression or non-inflammatory ischemia of the median nerve. This pathology occurs in 1.5–3% of the adult population and in 5% of specific risk groups (smoking people, people with obesity, rheumatoid arthritis, diabetes, hypothyroidism, multiple sclerosis) [1]. Carpal tunnel syndrome can occur at any age, but the peak incidence is between ages 40 and 60. CTS is more common in women (female:male ratio = 5:1) [2, 3]. The carpal tunnel syndrome is manifested by hypoesthesia, paresthesia, and pain in the affected area. These manifestations are followed by the thenar hypertrophy and a sharp decrease in hand function.

The CTS treatment includes non-operative procedures and surgical intervention. It could be cured completely if treated promptly. On the contrary, late treatment causes lesions and prolonged effects that seriously affect work and activities of daily living. Non-operative interventions (wrist splints and steroid injections) are assigned at the early stages of CTS. Although such interventions reduce CTS symptoms in a short time, they (symptoms) reoccur [4–6]. The carpal tunnel release (CTR) surgery, in which the surgeon cuts the transverse carpal ligament, is the most radical treatment, assigned in moderate and severe CTS (stages 2 and 3 by R. Szabo [7]) or when non-operative treatment fails [5]. In Vietnam, open surgery is a common treatment for CTS that had been in use for years, while the endoscopic surgery is an innovation with many advantages (esthetically small scar surgery on wrist, painlessness and faster recovery time) [8–11]. The endoscopic carpal tunnel decompression of the median nerve is a common world practice now. In our opinion, it is a promising surgical treatment of patients with CTS, mostly moderate, in Vietnam.

The purpose of this study is to evaluate the outcomes of single-portal endoscopic carpal tunnel release surgery in Hanoi Medical University Hospital, to assess the efficacy of this method and the prospects of introducing it as a surgical treatment option for patients with CTS in Vietnam.

## Methods

This is a prospective study conducted on 150 hands in 118 patients diagnosed with CTS and assigned to endoscopic surgery in Hanoi Medical University Hospital. Some patients underwent ultrasonography and electromyography (EMG) in Hanoi Bach Mai University Hospital and Hanoi VietDuc University Hospital. The study period is from May 2016 to December 2017. *Inclusion criteria* for surgical treatment were the common symptoms of CTS: nocturnal acroparesthesia, hypoesthesia at the hand dermatome, reduced strength in the hand, positive Tinel’s and Phalen’s signs, signs of compression of the median nerve on ultrasound, median nerve cross-sectional area of 13–15 mm^2^, reduced amplitude of a tenor muscle response and latency elongation. Surgical treatment was suggested in the absence of effect from a drug therapy. *Exclusion criteria* include the following*:* gout in the wrist, wrist lumps, proximal median neuropathy, cervical radiculoneuropathy, history of wrist surgeries, and carpal tunnel injuries.

The following provocative tests were used in the diagnosis of CTS: Wrist flexion test (Phalen’s test), carpal compression test (Durkan test), Tinel’s percussion test, and two-point discrimination test [12]. Diagnostic criteria for CTS include numbness and tingling in the median nerve distribution, nocturnal numbness, and weakness and/or atrophy of the thenar musculature [13]. All patients underwent electroneuromyography on a Keypoint Dantec 8. The electroneuromyography protocol implies examination of the motor and sensory responses of a median nerve (latency, amplitude, and conduction velocity). Diagnostic parameters included a decreased conduction velocity along the sensory nerve fibers in the palm (< 50 m/s), prolonged distal motor latency (> 4.0 ms) and S-wave latency (> 3.5 m/s at fixed 12-cm distance between the stimulator and the recording electrode), and decreased amplitude of *M* (< 4.5 mV) and *S* (< 15 mV) responses [14].

Ultrasound imaging was performed on all patients using a high-performance LOGIQ 9, a device that offers a high-resolution color monitor providing images without any flicker (General Electric, USA). The scanner is equipped with a broadband linear sensor with a frequency range of 11–14 MHz. The cross-sectional area of the median nerve was measured at different segments (the forearm, the entrance of the carpal tunnel, the carpal tunnel, the exit of the carpal tunnel, the palm). The CTS was diagnosed when the flattening ratio (FR) amounted to not less than 3.0 mm at the level of the distal carpal tunnel. This diagnosis was also made when the nerve at the proximal and the distal site of compression became thicker, although the cross-sectional area remained over 10 mm^2^. The abnormal median nerve CSA at the scaphoid-pisiform level can range 10–13 mm^2^ (mild expansion value), 13–15 mm^2^ (moderate expansion values), and 15 mm^2^ (severe expansion value) [15–17]. The nerve expansion values match the corresponding neurophysiological grades of CTS.

The patients were followed up clinically using the Boston questionnaire (BQ), electromyography, and ultrasound imaging at 1 month, 3 months, and 6 months postoperatively.

To measure CTS severity, this study uses a clinical severity scale denoted a “Hi-Ob” scale. There are five Hi-Ob scores of increasing severity: “1”—only nocturnal paresthesia, “2”—diurnal paresthesia, “3”—sensory deficit, “4”—hypotrophy and/or motor deficit of median innervated thenar muscles, and “5”—complete atrophy or plegia of median innervated thenar muscles [18].

### Surgical methods

**Fig. 1:**
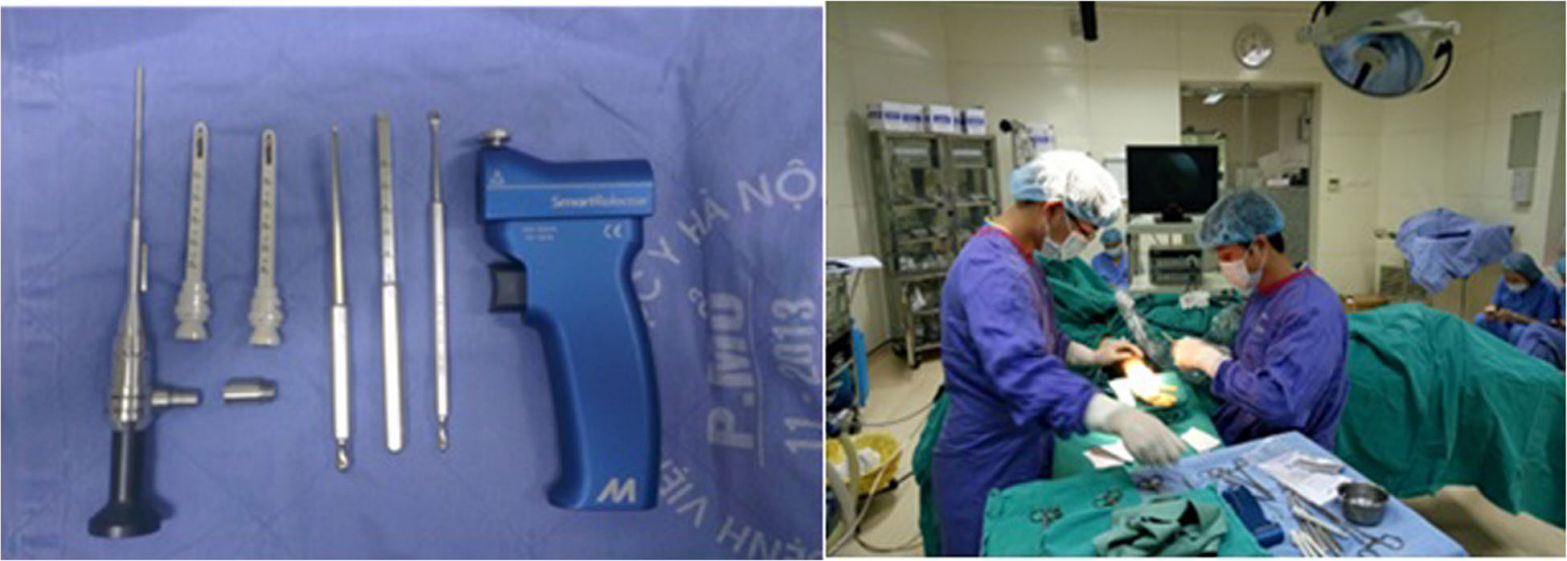
Surgical instruments and patient’s position

Preparation: Basic hand surgery set, endoscopic carpal tunnel release system (MICROAIRE) with a 3.0-mm eyepiece endoscope connected to a standard camera connector, camera, light source, and pneumatic tourniquet. Operations were performed under block and local anesthesia with the use of 20 ml of 1% solution of marcaine Fig. 1.

**Fig. 2:**
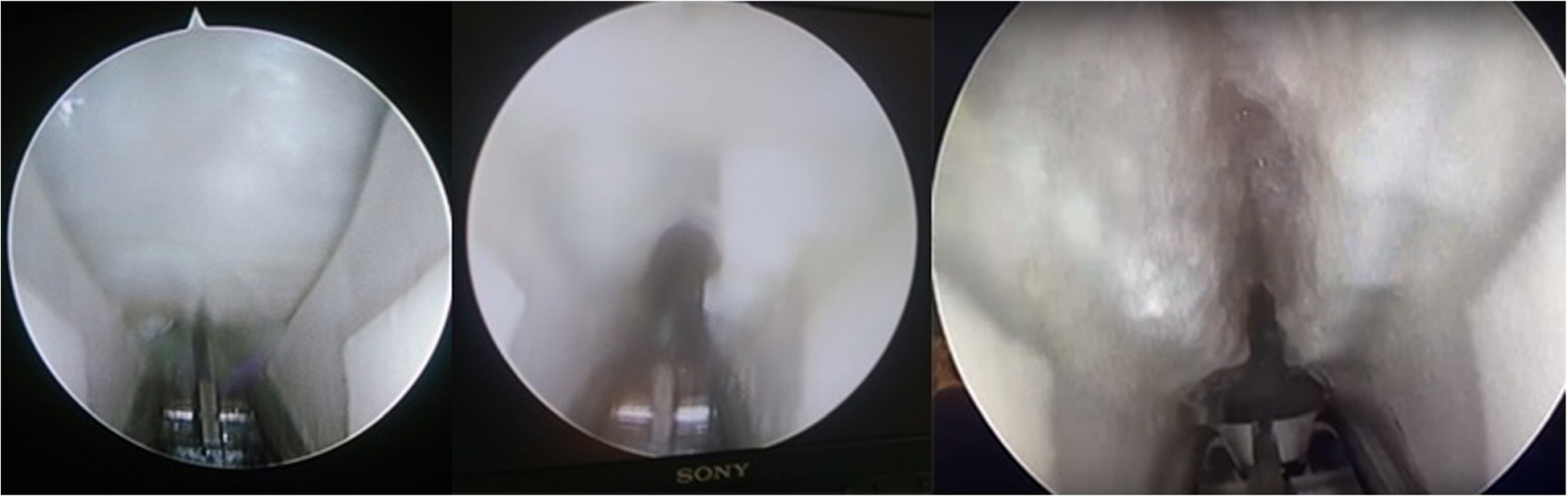
Transverse carpal ligament, cut and completely divided

Agree’s endoscopic CTR surgery procedure, with the placement of a MicroAire system:

- A single 1.0–1.5-cm incision is made transversely at the level of the proximal palmar wrist crease. Palmaris longus tendon is retracted radially.
- The carpal tunnel is dilated with dilators, aligned with the base of the ring finger, and then a slotted cannula is placed into the carpal tunnel. The transverse carpal ligament is divided from the distal with the blade under direct vision of the endoscope. Note: the cannula should not be placed too deep into the tunnel as this may cause damage to the superficial palmar arch, which is 3.5 cm distal to the distal palmar wrist crease.
- The wound is closed in a single layer with sutures and bandaged tightly. The wrist is immobilized in a splint for 2 weeks Fig. 2.

## Study results

### General information about patients

- Study involved 150 hands in 118 patients, 20 men (17%), and 98 women (83%) with the average age of 51.7 years (range 40–60 years).
- Duration of symptoms before surgery averaged 22.9 ± 4.8 months (range 6–84 months).
- Patients all had numbness in their palms and fingers (100%). Some patients had paresthesia (37.3%), wrist pain (26%), and weakness (21.3%).
- Phalen’s test was positive for 92% of hands, Durkan test—for 88%, Tinel’s test—for 64%. Thenar atrophy occurred in 40 hands (26.7%).
- *Two-point discrimination test* revealed abnormal skin sensation with slight to severe reactions (4% slight reactions, 34% moderate reactions, 32% serious reactions, 30% severe reactions).
- Fifty-two percent of patients had their right hands operated, and the remaining 48% patients had their left hands operated (32 patients had both hands operated).
- One hundred and two hands (68%) were dominant.
- Median nerve CSA on ultrasound averaged 16.7 mm^2^.

### Treatment outcomes

In this study, intraoperative complications did not occur. There were no cases needing open surgery during the operation. Recurrent symptoms were not detected at the follow-up Tables 1 and 2.

**Table 1:** Recovery from numbness

Result	1 month		3 months		6 months	
	*N*	%	*N*	%	*N*	%
Recovered	0	0	48	32.0	138	92.0
Improved	147	98.0	102	68.0	12	8.0
Not improved	3	2.0	0	0	0	0
Total	150	100	150	100	150	100

**Table 2:** Post-operative scores for BQ

Time	Mean ± SD	Min–max	*p*
Pre-operative	3.43 ± 0.59	2.51–4.31	
1 month postoperatively	2.43 ± 0.48	1.49–3.24	0.000
3 months postoperatively	1.82 ± 0.38	1.23–2.73	0.000
6 months postoperatively	1.30 ± 0.33	0.71–2.15	0.000

At 6-month follow-up, BQ score fell from 3.43 ± 0.59 to 1.30 ± 0.33. The difference is statistically significant at *p* < 0.05.

In the pre-operative period and 6 months after surgery, the number of hands with normal *two-point test* values (patients should be able to recognize two-point separation of 8–12 mm on the palms [12]) significantly increases from 0% to 96%. After 6 months, only 4% of hands remained with slightly abnormal skin sensation Tables 3 and 4.

**Table 3:** Post-operative clinical tests

	Tinel’s test (+)		Phalen’s test (+)		Durkan test (+)	
	*N*	%	*N*	%	*N*	%
Pre-operative	96	64.0	138	92.0	132	88.0
1 month postoperatively	21	14.0	102	68.0	48	32.0
3 months postoperatively	0	0.0	12	8.0	0	0.0
6 months postoperatively	0	0.0	0	0.0	0	0.0

**Table 4:** Post-operative muscular atrophy

	*N*	%
Pre-operative	40	26.7
1 month postoperatively	40	26.7
3 months postoperatively	30	20
6 month postoperatively	21	14

Muscular atrophy reduced from 26.7% to 20% by the 3-month follow-up and to 14% by the 6-month follow-up Table 5.

**Table 5:** Post-operative median nerve electroneuromyography

	DMLD	DSLD
Pre-operative	3.33 ± 0.35	0.91 ± 0.13
1 month postoperatively	3.03 ± 0.31	1.69 ± 0.11
3 months postoperatively	2.21 ± 0.15	1.31 ± 0.10
6 months postoperatively	1.10 ± 0.15	0.70 ± 0.08

- *Median ulnar motor latency difference* decreased gradually from 3.33 ms to 1.10 ms by the 6-month follow-up, *p* < 0.05).
- *Median ulnar sensory latency difference* decreased gradually from 0.91 ms to 0.70 ms by the 6-month follow-up, *p* < 0.05.

## Discussion

This study was conducted on 150 hands in 118 patients, 98 women (83%), and 20 men (17%). This ratio also corresponds to [5, 6, 19, 20]. CTS was commonly found in middle-aged women, probably due to hormonal changes.

The average duration of symptoms before surgery was 22.9 months (range 6 to 84 months). The majority of patients unsuccessfully underwent non-medical treatment before taking part in the study. Some patients were not diagnosed with CTS until late stages. This resulted in late treatment and consequently, in worse outcomes and slower recovery. Frédéric Schuind was dealing with symptoms that lasted even longer—25.6 months [21]. Hansen mentioned shorter duration of this disease; patients sought medical attention early—the average duration of symptoms was 10 months (range 6–12 months). This is illustrative of the difference in the quality of education and health services between our and other countries [22]. Studies comparing the effectiveness of various surgical methods are contradictory in their conclusions. Larsen compared the procedures of open-access decompression using inclusion and endoscopic techniques. The results observed 4 months after the surgery were similar [23]. The Zuo’s meta-analysis showed no significant difference in results and in the level of postoperative complications between the open and endoscopic surgeries [24]. Kang (2013) noted that 34 (65%) out of 52 patients, who underwent bilateral decompression (with minimally invasive and endoscopic approaches), would prefer endoscopic surgery [25]. Many authors describe a low recurrence rate and faster recovery after endoscopic decompression of the median nerve [8–11]. Sayegh and Strauch described the long-term effect from open and endoscopic decompression. Although there was no significant difference between two approaches, the endoscopic treatment allowed an earlier return to work. Additionally, authors indicate a lower risk of scar tenderness and a greater risk of nerve injury following the endoscopic release [9]. According to Faucher, the risk of developing transient neurological disorders due to nerve injury is higher with endoscopic decompression than with the open procedure. However, the choice of an approach does not affect the risks of intraoperative nerve injury with the occurrence of persistent neurological deficit [26].

### Clinical

At 1-month follow-up, hands did not reach full recovery: 98% of hands showed improved numbness, paraesthesia, and pain, and only 2% of hands had no improvement. At 3-month follow-up, 32% of hands reached full recovery of the muscle function, while the remaining 68% still had issues.

However, at 6-month follow-up, full recovery of the muscle function was achieved by 92% of hands, while the remaining 8% showed some improvements but did not reach full recovery. Patients had their sleeping problems resolved and the motor function restored.

The BQ score fell from initial 3.43 to 1.30 by the time of the 6-month follow-up. This difference is statistically significant at *p* < 0.05. These results are similar to those in [8–11, 20, 27–31].

### Post-operative thenar muscles atrophy

- Before surgery, there were 26.7% of patients with muscular atrophy. At 6-month follow-up, their number fell to 14%. These patients were operated in the late stage of CTS due to a lack of early diagnosis or improper non-operative treatment.
- Different studies report on slow recovery, which lasts 12 months after surgical intervention [29, 32–34].

### Pre-operative median nerve on EMG

Motor latency difference between median nerve and ulnar nerve was 3.33 ms on average, 1.25 higher than the normal value [28, 35–37]. Sensory latency difference between median nerve and ulnar nerve was 0.91 ms on average, 0.79 higher than the normal value.

### Post-operative median nerve on EMG

- Median versus ulnar motor latency difference decreased gradually throughout the recovery period, from initial 3.33 ms to 3.03 ms at the 1-month follow-up and to 1.10 ms at the 6-month follow-up. Such a change is statistically significant at *p* < 0.05.
- Median versus ulnar sensory latency difference decreased from pre-operative 0.91 ms to 0.70 ms by the time of the 6-month follow-up. This difference is statistically significant at *p* < 0.05.
- EMG findings start showing significant improvement at 3-month follow-up onward. At the 1-month follow-up, electromyographic values do not change significantly due to a big negative value of pre-operative difference in sensory latencies.

## Conclusion

The endoscopic surgery provides good outcomes in patients. This relatively simple surgery improves hand function and sensation within a short recovery period. The findings of this study demonstrate encouraging results.

In this study, the endoscopic release using the microaire single-portal system happened to be a safe and efficacious option for endoscopic carpal tunnel release. In our opinion, it can be widely implemented in the surgical practice for the treatment of carpal tunnel syndrome, especially moderate cases (2 stage by R. Szabo) [7]. However, given the contradictions in the literature regarding the associated complications, the suggestion is to conduct further research on a larger number of surgical procedures for treating CTS of different severity and to conduct additional prospective studies.

## Abbreviations

BQ score: Boston questionnaire score
CSA: Cross-sectional area
CTS: Carpal tunnel syndrome
EMG: Electromyogram
Hi-Ob: History-objective
